# A High-Precision Mid-Infrared Spectrometer for Ambient HNO_3_ Measurements

**DOI:** 10.3390/s22239158

**Published:** 2022-11-25

**Authors:** Nicolas Sobanski, Béla Tuzson, Philipp Scheidegger, Herbert Looser, Christoph Hüglin, Lukas Emmenegger

**Affiliations:** 1 Empa–Swiss Federal Laboratories for Materials Science and Technology, Laboratory for Air Pollution and Environmental Technology, Überlandstrasse 129, 8600 Dübendorf, Switzerland; 2 Empa–Swiss Federal Laboratories for Materials Science and Technology, Transport at Nanoscale Interfaces, Überlandstrasse 129, 8600 Dübendorf, Switzerland

**Keywords:** air pollution, trace gas detection, nitric acid, mid-infrared absorption spectroscopy, quantum cascade laser

## Abstract

Precise and accurate measurements of ambient HNO_3_ are crucial for understanding various atmospheric processes, but its ultra-low trace amounts and the high polarity of HNO_3_ have strongly hindered routine, widespread, direct measurements of HNO_3_ and restricted field studies to mostly short-term, localized measurement campaigns. Here, we present a custom field-deployable direct absorption laser spectrometer and demonstrate its analytical capabilities for in situ atmospheric HNO_3_ measurements. Detailed laboratory characterizations with a particular focus on the instrument response under representative conditions for tropospheric measurements, i.e., the humidity, spectral interference, changing HNO_3_ amount fractions, and air-sampling-related artifacts, revealed the key aspects of our method: (i) a good linear response ($R^{2}$ > 0.98) between 0 and 25 nmol·mol${}^{-1}$ in both dry and humid conditions with a limit of detection of 95 pmol·mol${}^{-1}$; (ii) a discrepancy of 20% between the spectroscopically derived amount fractions and indirect measurements using liquid trapping and ion chromatography; (iii) a systematic spectral bias due to water vapor. The spectrometer was deployed in a three-week field measurement campaign to continuously monitor the HNO_3_ amount fraction in ambient air. The measured values varied between 0.1 ppb and 0.8 ppb and correlated well with the daily total nitrates measured using a filter trapping method.

## 1. Introduction

Reactive nitrogen oxides play a central role in tropospheric chemistry. They are involved in ozone (O_3_) formation [1,2], the nitrate (NO${}_{3}^{-}$) fertilization of soils via wet and dry deposition [3,4,5], and the formation of oxidized volatile organic compounds (VOCs) [6]. At ground level, reactive nitrogen is mainly emitted as NO_x_ (NO + NO_2_) due to the burning of fossil fuels (65% of global yearly emissions). The total amount of NO_x_ emissions was 50 TgN·yr${}^{-1}$ in 2014 [7,8]. NO_x_ is the precursor of numerous more oxidized forms of nitrogen oxides in the gas and particle phases (HNO_3_, HONO, NO_3_, N_2_O_5_, RONO_2_, NO${}_{3}^{-}$, etc.), which are usually referred to as NO_z_. The sum of NO_x_ and NO_z_, often labeled as NO_y_, represents the whole family of reactive nitrogen oxides.

The NO_x_-to-NO_y_ ratio in ambient air strongly depends on the proximity of NO_x_ emission sources (in terms of the transport time) and shows a negative gradient when moving away from urban/industrialized areas to rural/remote areas or to the upper troposphere. This decrease is due to the oxidation of NO_x_ to NO_z_ species by the hydroxyl radical (OH), O_3_, or organic radicals (RO and RO_2_). At some point in this process, the NO_z_ amount fraction starts to dominate that of NO_x_ [9,10]. This phenomenon is influenced by various factors (temperature, total radiation, humidity, VOCs, etc.), and, therefore, it shows significant daily, seasonal and spatial variability [6,9,11]. The progressive development of analytical measurement techniques for individual NO_y_ species has allowed a better understanding of the various NO_x_ oxidation pathways. This has highlighted the central role of nitric acid (HNO_3_) among reactive nitrogen oxides and its general importance for atmospheric chemistry.

The main HNO_3_ source in the atmosphere is the reaction between NO_2_ and OH, which accounts for 60% of direct, irreversible NO_2_ losses according to Stavrakou et al. [12]. The reaction between NO and HO_2_ has more recently gained attention as another significant source of atmospheric HNO_3_ [13,14]. These two reactions play a major role in the troposphere since they impact ambient HO_x_ (HO + HO_2_) amount fractions, which in turn influence the production of O_3_ and the oxidation of VOCs. The corresponding formation pathways are fast enough that the HNO_3_ amount fraction can dominate that of other NO_y_ species in photochemically aged air masses. This is often observed when VOCs are present in low concentrations [15]. Dry and wet deposition represents the main atmospheric sink for HNO_3_ and indirectly represents the strongest NO_x_ sink on a global scale [4,5].

Precise and accurate measurements of HNO_3_ for laboratory and field studies are crucial for better quantification of the various mechanisms mentioned above, and they are needed to support modeling studies. However, the ambient amount fractions of HNO_3_ (from nmol·mol${}^{-1}$ or part per billion by volume (ppb) down to the low pmol·mol${}^{-1}$ or part per trillion by volume (ppt) range) make this challenging. In addition to its low concentration, the high polarity of HNO_3_ results in its very rapid removal from the gas phase through adsorption on surfaces, which strongly constrains the design of analyzers. The adsorption/desorption processes often limit the instrument’s response time. All these aspects have hindered routine, widespread direct measurements of HNO_3_ and restricted field studies to mostly short-term, localized measurement campaigns [6,11].

Early measurements of HNO_3_ used indirect methods that rely on HNO_3_ capture by nylon filters or aqueous denuders, followed by the quantification of the trapped nitrate ions (NO${}_{3}^{-}$) [16,17,18]. With these methods, low limits of detection (LODs) (down to 120 ppt in 24 h [18]) can be achieved at the cost of temporal resolution, which limits the use of these methods for ambient air measurements. Indeed, photo-oxidative processes in the atmosphere happen on a time scale of hours to minutes and require a higher temporal measurement resolution to be efficiently observed. Furthermore, these methods are sensitive to the presence of other nitrogen oxides [19,20], which dissociate to unwanted nitrate ions and create a positive bias. Presently, the most used technique for in situ, direct measurements of HNO_3_ is chemical ionization mass spectrometry (CIMS) [21,22,23]. It offers high precision (down to 15 ppt at a resolution of 1 s), high selectivity, and a fast response time (down to a few seconds). However, the size and costs of such instruments hinder their use for widespread, routine measurements.

Direct laser absorption spectroscopy (LAS) in the mid-infrared (mid-IR) range has only rarely been used to measure ambient HNO_3_ amount fractions [24,25], despite its clear advantages over mass spectrometry. It requires, for example, less maintenance and less frequent calibration, and it is more easily deployed in field conditions. However, two important factors have often been considered to limit the use of LAS for HNO_3_ measurements, namely, the precision and response time.

The precision of laser spectrometers is strongly dependent on two parameters: the optical path length (OPL) and the absorption line strength. For HNO_3_, the latter is significantly lower than it is for other atmospherically relevant trace gases such as NO_2_, CO_2_, or CH_4_. To be able to detect low amount fractions, the weak line strength needs to be compensated for by increasing the OPL. This approach, however, does not necessarily lead to an improved signal-to-noise performance beyond a certain limit [26]. Furthermore, it usually results in large-volume absorption cells that have a negative impact on the response time of the instruments. To improve this factor, fluorinated silane or Teflon-like coatings have been used to increase the chemical passivity of the frequently used pyrex optical cells [24]. More recently, Roscioli et al. [27] obtained a response time of a few seconds by using an active passivation method that relies on the continuous injection of perfluorinated organic acid vapor into the sample flow. This achievement is, however, counterbalanced by the increased complexity of the inlet design and the need for additional consumables.

Although the abovementioned studies have shown that HNO_3_ can be quantified using a variety of methods, there is still a lack of routine measurement solutions. In order to fill this gap, we present a field-deployable quantum cascade laser absorption spectrometer (QCLAS) for ambient HNO_3_ measurements for very low amount fractions (down to tens of ppt). The key element of the instrument is a custom multipass cell (MPC) that allows both a high precision and a fast response time. We focused our efforts on the characterization of the instrument’s LOD, response time, and linearity. We also investigated the influence of water vapor on the HNO_3_ measurements in representative conditions for tropospheric measurements. For quantitative validation, a permeation device was used to generate highly stable amounts of HNO_3_ in the 0–25 ppb range. The calibration of the permeation source was performed by nitrate liquid trapping coupled with ion chromatography. In addition to these laboratory investigations, the spectrometer was used in a three-week field measurement campaign at a rural site in central Switzerland in September 2021.

## 2. Materials and Methods

### 2.1. QCLAS Setup

The design of the HNO_3_ spectrometer is based on a previous instrument initially developed for NO_x_ measurements that is described in detail elsewhere [28]. Briefly, the optical setup includes a distributed feedback (DFB) quantum cascade laser (QCL; Alpes Lasers, St. Blaise, Switzerland) mounted in a custom-made water-cooled housing with an embedded thermoelectric-controlled (TEC) heatsink and collimation optics. The laser is operated at −10 °C. The excess heat is dissipated by a thermochiller (T-Three, Solid State Cooling System, Wappingers Falls, NY, USA). The laser beam is coupled into a custom-designed astigmatic Herriott MPC using a series of beam shaping and steering mirrors. The transmitted light is measured by a HgCdTe (MCT) photovoltaic detector (PVI-4TE-6, VIGO Photonics, Ożarów Mazowiecki, Poland). The data acquisition system, as well as the laser driver electronics, are custom-made solutions [29] based on a commercial programmable board (ALPHA250, Koheron); they are built around a field-programmable gate array (FPGA; Zynq 7020, Xilinx, Inc., San Jose, CA, USA). This unit features 100 MHz low-noise RF front-ends with 14-bit ADCs and 16-bit DACs at 250 MS/s. A custom-written FPGA program triggers, reads, and controls the peripherals (e.g., the TECs, valves, mass flow controllers (MFCs), and temperature and pressure sensors). The QCL is driven in iCW mode [30,31], i.e., current is periodically applied to the laser with a repetition rate of 10 kHz, creating an emission frequency sweep of 0.6 cm${}^{-1}$ centered around 1720.6 cm${}^{-1}$. This range was chosen because it contains strong HNO_3_ absorption lines of the $\nu_{2}$ band, with very low spectral interference from other atmospheric compounds and especially from water vapor.

**Figure 1:**
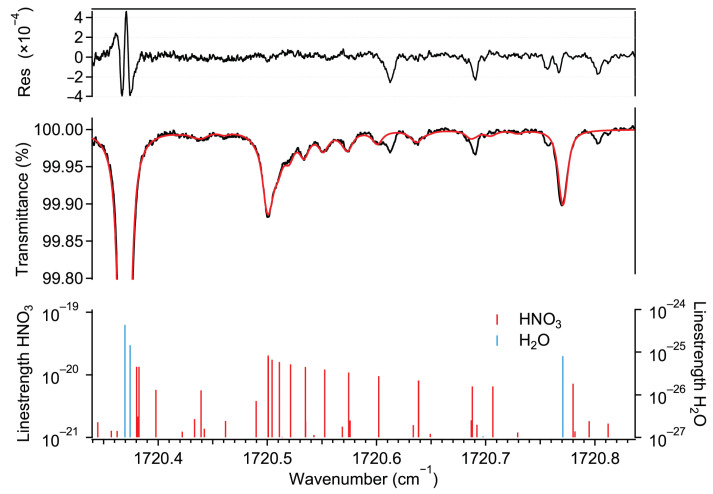
**Bottom**: ro-vibrational transitions of HNO_3_ and H_2_O taken from HITRAN2020 [32]. **Middle**: measured transmission spectrum (black) of a gas mixture (30 °C, 5 kPa) of ambient air and HNO_3_-containing air and the associated spectral fit using the Voigt profile (red). The retrieved amount fraction corresponds to 12 ppb HNO_3_ and 1.6% H_2_O, respectively. **Top**: fit residual indicating the quality of the fit (root mean square of the fringe level of $2.5\times 10^{-5}$) and the existence of some absorption features that could not be associated with any known transition listed in HITRAN2020.

Figure 1 shows a typical transmission spectrum recorded by our instrument, along with a least-squares Voigt profile fit for the HNO_3_ and H_2_O absorption lines using the molecular parameters from the HITRAN2020 spectral database [32]. A total of 25 transitions (mainly doublets) were considered to fit the absorption features of HNO_3_. The transmission spectrum corresponds to 12 ppb HNO_3_ and an absolute humidity (abbreviated as % H_2_O in the rest of the text) of 1.6%. This spectrum was obtained by averaging 10${}^{3}$ traces (corresponding to a 1 s acquisition time) at 25 °C and at a sample pressure of 5 kPa. The fit residual reveals some absorption features with amplitudes significantly higher than the noise level. These could not be identified using the spectral database. The presence of the water absorption lines within the tuning range makes it possible to determine the absolute frequency axis even in the case of very low ambient HNO_3_ amount fractions.

The MPC ensures an OPL of 110 m, corresponding to 274 reflections between its mirrors, which are separated by a distance of 40 cm and have diameters of 8 cm [28]. The entire cell body (aluminum) was surface-treated to mitigate the adsorption of reactive species on its inner walls. This surface treatment consisted of electro-polishing, which was followed by coating the surface with a SilcoNert^®^2000 (SilcoTek GmbH, Bad Homburg, Germany) layer. This coating, based on hydrogenated amorphous silicon, has been widely used to improve the sampling efficiency of chemically active analytes in a variety of measurement instruments, for example, for measuring ammonia (NH_3_) [33]. Furthermore, the MPC was insulated and thermally stabilized at 30±0.1 °C.

The inlet of the instrument, optimized to minimize the adsorption of HNO_3_, is described in the following. The low pressure (5 kPa) in the MPC is obtained by compressing a small section of the PFA 1/4-inch tube upstream of a PTFE membrane filter (TE38, Cytiva Europe GmbH, Freiburg im Breisgau, Germany). Downstream of the filter, a Teflon 3-way solenoid valve (International Polymer Solutions, Irvine, CA, USA) makes it possible to switch between ambient air (sampling channel) and HNO_3_-scrubbed air (zeroing channel; see the paragraph below). The pressure drop (about 5 hPa) created by the scrubber (NaHCO_3_-impregnated nylon wool housed in a glass cartridge) is compensated for on the sampling channel by an additional flow restriction on the PFA 1/4-inch tube. Both channels are connected immediately before the MPC inlet by a PFA T-piece. A continuous gas flow is maintained by a scroll pump (nXDS15i, Edwards Vacuum, West Sussex, UK) at a normalized flow rate of 8.5 slpm, corresponding to an effective flow rate of about 170 L·min${}^{-1}$ through the instrument.

The presence of water vapor in the sample during ambient air measurements can lead to spectroscopic biases in the retrieval of the HNO_3_ concentration [28,34]. This is often the case if dry air is used in the blank measurements to correct for offset drifts. To minimize this effect, we use HNO_3_-free ambient air at ambient humidity instead of dry N_2_. This is achieved by chemically removing HNO_3_ from the sample gas using NaHCO_3_-impregnated nylon wool [21,35].

### 2.2. Experimental Setup for Laboratory Characterization

**Figure 2:**
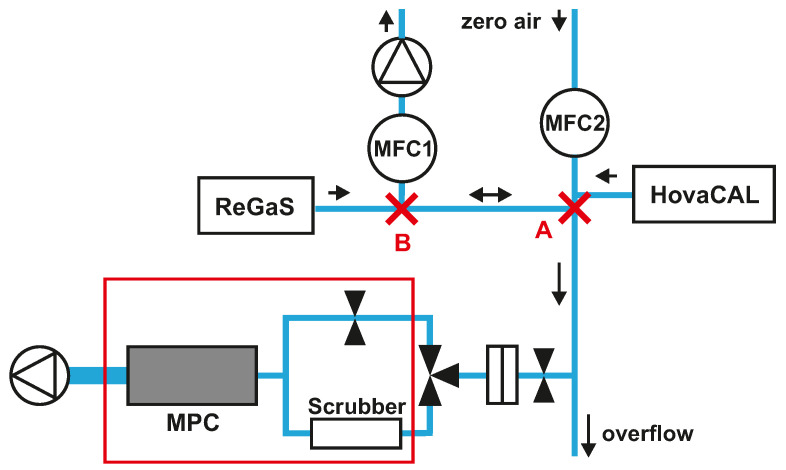
Laboratory setup for characterization experiments. The red frame indicates the elements embedded in the housing of the spectrometer. A: the position where the step-wise variation of HNO_3_ is applied for inlet response time experiments (see text). B: the position where the share of HNO_3_-containing air in the calibration gas mixture is controlled. Arrows indicate the flow direction.

A schematic of the spectrometer gas sampling system and of the calibration setup used for laboratory measurements is depicted in Figure 2. Stable amounts of HNO_3_ are generated using a permeation instrument (ReGaS) developed by the Swiss Metrology Institute (METAS) and described in Pascale et al. [36]. During operation, the HNO_3_ permeation tube is permanently flushed with 0.3 slpm of zero dry air. This HNO_3_-containing airflow is then diluted internally to match the target HNO_3_ gas content. For the laboratory experiments, the ReGaS output concentration was kept constant (at about 60 ppb; more details in Section 3.1) at a flow of 3.5 slpm. A mass flow controller (MFC1) is used to vary the amount fractions of HNO_3_ in the calibration gas without the need to change the ReGaS set point, thereby avoiding the very long response time (up to a few hours) of the internal ReGaS dilution system. This is achieved by removing a portion of the ReGaS output flow. A second mass flow controller (MFC2) is used to inject zero air into the calibration line and bring the total calibration gas flow rate to at least 9 slpm, thus ensuring a minimum overflow of 0.5 slpm. With this setup, the amount fraction of HNO_3_ at the instrument inlet (point A) can be varied between 0 and 25 ppb by adjusting the MFC1 set point between 3.5 and 0 slpm. To generate stable amounts of water vapor of up to 1.2% H_2_O, a hot-vapor-calibration (HovaCAL, IAS GmbH, Oberursel, Germany) system is used.

The main advantage of this setup is that it allows quasi-instantaneous switching between a given HNO_3_ amount fraction and zero HNO_3_ at the instrument’s inlet. This is achieved by setting MFC1 to a value greater than the output of the ReGaS, thus reversing the flow direction between points A and B of the calibration line. This feature was used to determine the response time of various elements of the sampling inlet (see Section 3.1.1).

The uncertainty in the permeation rate, the purity of the permeation tube, and the internal transmission efficiency of the ReGaS do not allow it to be used directly as a calibration standard. Therefore, the generated amount fraction was measured by letting the output gas stream flow through a bubbler, trapping HNO_3_ in water in the form of nitrate ions (NO${}_{3}^{-}$). PFA tubing was used to bring the gas sample from the ReGaS to the fritted glass part of the bubbler to avoid permanent losses of HNO_3_ on the glass labware surfaces. After a few hours, the gas flow was stopped and the nitrate-containing water was analyzed offline using ion chromatography (Dionex Aquion IC System, Thermo Fisher Scientific, Inc., Waltham, USA), as described in Huey et al. [21]. Before every such experiment, the trapping system was passivated for at least 12 h to reach a gas phase/surface equilibrium.

### 2.3. Field Deployment

#### 2.3.1. Measurement Site and QCLAS Inlet Setup

**Figure 3:**
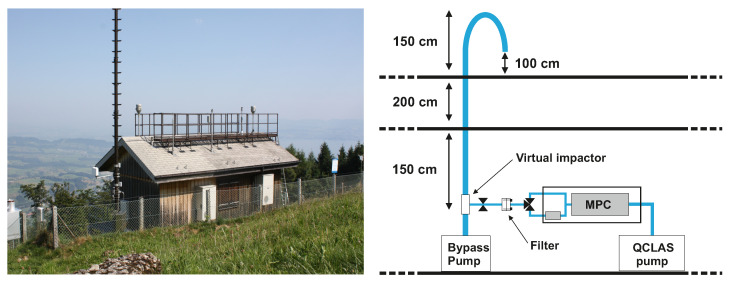
**Left**: Rigi measurement station as seen from the southeast. **Right**: schematic of the inlet and sampling system.

The QCLAS was deployed during a three-week measurement campaign in September 2021 at a rural site in Switzerland. The measurement site (600 m above the surrounding plateau, 1300 m above sea level, Figure 3) is an air quality monitoring station that is part of the Swiss Air Quality Monitoring Network (NABEL) and is located on the northwestern slope of the mountain Rigi-Kulm, 10 km to the east of the city of Luzerne (80,000 inhabitants). The station’s surroundings are pre-alpine, with a mixture of forest and pastoral lands. The region consists of a mixture of sparsely populated urban and agricultural areas.

The sampling system used for the field deployment is shown in Figure 3. The air intake was situated 1 m above the rooftop using a 6.5 m long PTFE pipe with an inner diameter of 22 mm. The tube tip was bent to prevent rain from dropping into the inlet. The air intake was situated 1 m above the rooftop. A custom-made PTFE virtual impactor (estimated cut-off of about 2.5 μm) removed coarse particles from the sampled ambient air. To achieve this, the inlet flow was accelerated through a 4 mm nozzle. A side flow of 8.5 slpm was directed to the QCLAS, while the remaining flow of 30 slpm was used to entrain the coarse particles.

During the field campaign, a zero-drift-correction routine was used to improve the measurement performance of the HNO_3_ spectrometer (see Section 3.1.1). This routine, which involved the periodic measurement of dry and humid HNO_3_-free air, is as follows: every 2 h and for 5 min, 10 slpm of zero air was injected (using an MFC) into the sampling line via a T-piece connector positioned between the virtual impactor and the first pressure reduction of the spectrometer inlet. This made it possible to obtain a blank for H_2_O measurements. Within this 2 h cycle, an HNO_3_ blank was obtained every 15 min by switching the solenoid 3-way valve between the sampling and zeroing channels. During this 15 min cycle, air flowed through the sampling channel for 10 min and then through the zeroing channel for 5 min. The first 2 min of data after the valve switching were discarded to account for the MPC response time. The drifts in both the H_2_O and HNO_3_ zero levels were compensated for by linearly interpolating the average signal during the blank measurements and then subtracting it from the measured ambient data.

#### 2.3.2. Ancillary Measurements

The total nitrates (gas phase HNO_3_ and particle phase NO${}_{3}^{-}$), hereafter ΣNO${}_{3}^{-}$, are measured routinely at the Rigi station using a filter-based sampling method. Ambient air is actively pumped through the NaHCO_3_-impregnated nylon filter at a flow rate of 13 slpm. The sampling setup includes 14 filters in separate filter holders and a manifold valve that makes it possible to automatically switch between the filters every 24 h. All 14 filters are exchanged every 2 weeks (i.e., after every filter was used for one day), and the filters are brought to the laboratory, where trapped NO${}_{3}^{-}$ ions are measured using ion chromatography (Dionex Aquion IC System, Thermo Fisher Scientific, Inc.). The LOD for the total nitrates is 0.01 μgN·m${}^{-3}$, and the total uncertainty is 0.13 μgN·m${}^{-3}$.

The gas phase HNO_3_ can be estimated based on ΣNO${}_{3}^{-}$ measurements using various thermodynamic equilibrium models, including the ISORROPIA II model that we used in this study [37]. This model requires further parameters to calculate the gas phase HNO_3_, including the total ammonium (gas phase NH_3_ and particle phase NH${}_{4}^{+}$) and total sulfate. The total ammonium (ΣNH${}_{4}^{+}$) is obtained using the same method that is used for the total nitrates, except that trapped NH${}_{4}^{+}$ ions are measured (also using ion chromatography). The LOD and uncertainty of this measurement method are 0.10 μgN·m${}^{-3}$ and 0.45 μgN·m${}^{-3}$, respectively. Finally, the total sulfate in PM10 (sampled using a high-flow filter-based system) is measured using ion chromatography, with an LOD of 0.002 μgS·m${}^{-3}$ and a relative uncertainty of 10%.

NO_2_ is measured using two different instruments: a chemiluminescence detector equipped with a molybdenum converter (Mo-CLD, 42i-TL, Thermo Scientific Inc., Reinach, Switzerland), and a cavity attenuated phase shift spectrometer (CAPS; T500U, Teledyne API, San Diego, CA, USA). The CLD has an LOD of 150 ppt and a total measurement uncertainty of 6%. The CAPS uses blue light absorption around 500 nm to directly measure NO_2_; this method has an LOD of 40 ppt and a measurement uncertainty of 5%.

During the measurement campaign, the NO_2_, PM10, and QCLAS instruments sampled from three different inlets that have air intakes located about 1 m above the station rooftop, with a mutual horizontal distance of less than 2 m. Therefore, they were considered to be collocated. The inlet of the ΣNO${}_{3}^{-}$ and ΣNH${}_{4}^{+}$ sampling systems is located on the downhill-facing side of the measurement station, 2 m above the ground.

## 3. Results and Discussion

### 3.1. Characteristics of the QCLAS

#### 3.1.1. Limit of Detection and Response Time

The detection limit of the spectrometer depends mainly on two factors: the spectroscopic precision and the zero-drift-correction procedure. The first factor can be well estimated by the Allan–Werle deviation method [38], while the drift-correction procedure is mainly determined by the target measurement data coverage (duty cycle) and the response time of the instrument. Both the precision and the influence of the zero-drift-correction procedure are explored in more detail below.

**Figure 4:**
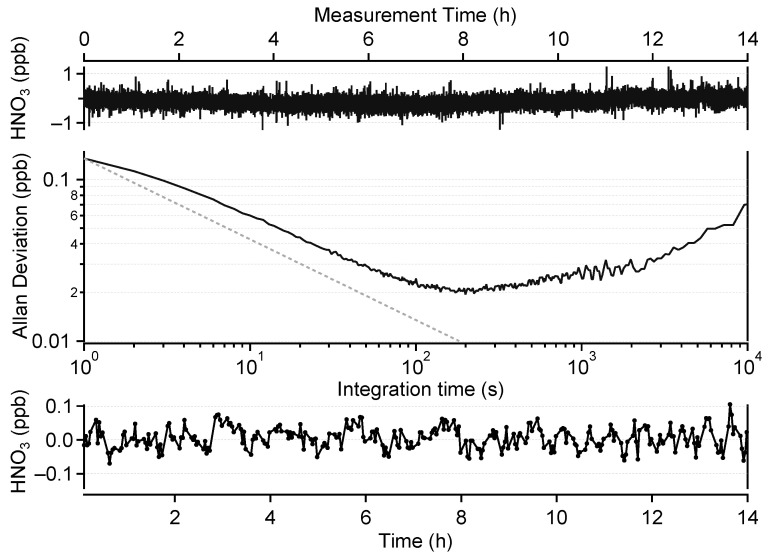
**Top**:HNO_3_-free air measurement with a resolution of 1 s over 14 h. **Middle**: corresponding Allan–Werle deviation plot. **Bottom**: 2 min averaged zero-drift-corrected data.

The top panel of Figure 4 shows a 14 h measurement of HNO_3_-free air under laboratory conditions. We follow this approach to eliminate the influence of the calibration source instability on the measured data. The Allan–Werle deviation plot associated with this time series (middle panel of Figure 4) shows 1 s and best achievable precisions (corresponding to an integration time of 200 s) of 140 ppt and 20 ppt, respectively. This compares well with previously published data. Horii et al. [24] reported a precision between 150 and 200 ppt at a resolution of 1 s with a 210 m OPL multipass cell and reached a precision of 45 ppt at an integration time of 40 s. McManus et al. [39] obtained a 1 s precision of 600 ppt with a 76 m OPL cell. Hence, the precision of our instrument, normalized by the OPL, offers an improvement of at least a factor of 2.

While the Allan–Werle deviation method makes it possible to estimate the short-term precision of the spectrometer, long-term drifts of the zero signal, due to optical misalignment and/or thermo-mechanical stress, can also impact the measurement performance. A common approach to accounting for such drifts is to frequently re-measure the zero level of the instrument (blank measurement) and apply a drift correction to the data in a post-processing step. Obviously, more frequent blank measurements make it possible to obtain a better representation of the zero drift and therefore, a better effective precision, at the cost of data coverage.

This correction method (described in Section 2.3.1) was implemented for the field data presented in Section 3.2. In order to evaluate its impact on the instrument performance, this method was also applied to the data shown in the top panel of Figure 4. Since these data were obtained using only zero air, an artificial classification of ambient and blank measurements was performed as follows: a cycle of 15 min was considered, including 8 min of ambient data and 3 min of blank data. Between segments, 2 min of data were considered to be “rejected” to mimic the transition period after valve switching. This yielded an effective data coverage of about 50%. After the drift correction, only data categorized as ambient data were kept and averaged over 2 min, which corresponds to the minimum of the Allan deviation curve. The resulting time series is plotted in the bottom panel of Figure 4. The standard deviation (1σ) of this dataset is 32 ppt. The 2 min LOD of our instrument is therefore estimated to be 96 ppt (3σ) for 50% data coverage.

The other critical factor for the measurement performance is the response time of the instrument. It limits both the frequency of the blank measurements and the observation of the vast amount of fraction variations of the analyte. Polar molecules in particular tend to be adsorbed/desorbed by surfaces, thus leading to memory effects. This is seen in the instrument’s response when the analyte amount fraction at the inlet is modulated in a step-wise fashion. We focus below on estimating the HNO_3_ response time and voluntarily omit the case of H_2_O. The rationale for this is that the response time of our instrument for H_2_O is negligible compared to the HNO_3_ case and does not constrain the instrument design and data processing routine.

**Figure 5:**
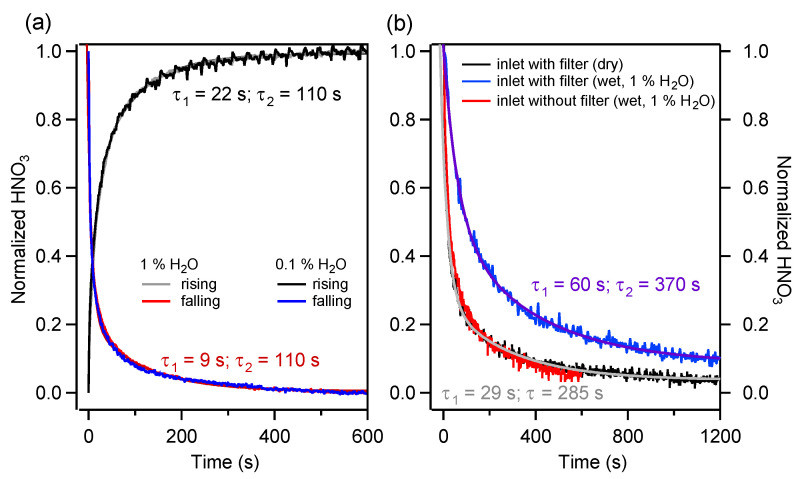
(**a**) Normalized response of the instrument’s reduced inlet to a step-wise HNO_3_ concentration change after a 3-way position switch in dry and humid conditions for both rising and falling edges. (**b**) Normalized response of the extended inlet of the instrument to a step change of the HNO_3_ concentration. Three configurations are shown: the response with a filter in dry (black) and humid (blue) conditions, and the response without a filter in humid conditions (red).

As the sampling line contains multiple elements, it is crucial to identify the most critical part in order to improve the overall response. We start by estimating the response times of the parts of the sampling system that are involved when switching the three-way valve position to perform HNO_3_ blank measurements. These parts are the MPC, the PFA piping of the ambient and zeroing channels, and the three-way valve itself. For more clarity, this part of the sampling system is designated the “reduced inlet” in the rest of the text. In Figure 5a, the HNO_3_ signal recorded after a valve position switch, normalized to unity, is shown. The experiment was conducted with an inlet HNO_3_ amount fraction of about 15 ppb and at 0.1% and 1% H_2_O. Each trace was obtained by averaging the results of triplicate measurements. The choice to make measurements at low but non-zero humidity was motivated by the need to lock the laser emission frequency even when measuring HNO_3_-free samples.

The rise and fall times of the HNO_3_ were found to be best described by a double exponential, with $\tau_{1}$ ∼ 22 s (for rising) and ∼9 s (for falling), and $\tau_{2}$ ∼ 110 s for both cases. Similar behavior has been observed also by Roscioli et al. [27]. Figure 5a shows two important facts: first, humidity does not affect the absorption–desorption kinetics of the investigated part of the sampling system, and second, the rise and fall times $\tau_{1}$ are significantly different. This can be directly related to the adsorption–desorption phenomenon (e.g., to different kinetic regimes resulting from the unequal values of the absorption and desorption reaction rate constants).

The response time of the entire instrument’s inlet (hereafter, “extended inlet”) has also been measured, due to its relevance for field measurement quality. The extended inlet consists of the reduced inlet plus the filter element and the PFA piping upstream and downstream of the initial pressure reduction element. For all experiments discussed and described in the following paragraphs, a step-wise variation (from 15 to 0 ppb) of the HNO_3_ amount fraction in the sampled gas mixture was applied at point A of the calibration line. This was achieved by changing the flow through MFC1 from 0 to 4 slpm (see Section 2.1). The PFA tubing length between point A and the pressure reduction element of the extended inlet was adjusted (to about 30 cm) to correspond to the tube length between the virtual impactor and the pressure reduction element during the field deployment (see Section 2.3).

Figure 5b shows the normalized HNO_3_ response of the extended inlet for dry and humid conditions (1% H_2_O). The fall time $\tau_{2}$ in dry conditions is about 5 min, i.e., three times longer than the fall time of the reduced inlet (Figure 5a). Furthermore, at 1% H_2_O, the fall time increases up to 6 min. Additionally, the $\tau_{1}$ shows a factor two increase. This behavior contrasts strongly with the results obtained for the reduced inlet, for which no dependency on the sample humidity was observed.

Complementary experiments were conducted with inlet design variations to determine the contributions of the various extended inlet elements to the response time. It was found that the largest impact was caused by the filter, as shown in Figure 5b by the contrast between the blue (inlet with the filter) and red (inlet without the filter) traces. Furthermore, we attribute the difference in the response time between the reduced inlet and extended inlet without the filter in dry conditions to the PFA tubing portion upstream of the pressure reduction element. No contributions from the PFA tubing on the low-pressure side were observed.

These findings make it possible to optimize the inlet system according to the response time. Ideally, the inlet should include a critical orifice for pressure reduction as early as possible in the sampling line. Sampling through PFA tubing at ambient pressure, even at high mass flow rates (up to 10 slpm), should be avoided.

#### 3.1.2. Linearity and Accuracy

In addition to adsorption (or physisorption), absorption (or chemisorption) can cause losses of the analyte in the gas stream. This phenomenon has been demonstrated for HNO_3_ by Neuman et al. [35], who showed that sampling HNO_3_-containing air through glass or metallic surfaces caused irreversible HNO_3_ losses of up to 80%. In the case in which the kinetics of the chemisorption reaction depend on the sampled HNO_3_ amount fraction, this phenomenon can cause a non-linear effect.

We investigated this effect by measuring varying HNO_3_ amount fractions between 0 and 25 ppb in various carrier gases: ambient laboratory air, dry zero air, and humid zero air. For the humid zero air and ambient air dilution experiments, additional dry zero air was added to the system using MFC2 to compensate for variations in the flow through MFC1. In this way, the share of humid or ambient air in the final gas mixture was kept constant while the HNO_3_ amount fraction was varied.

**Figure 6:**
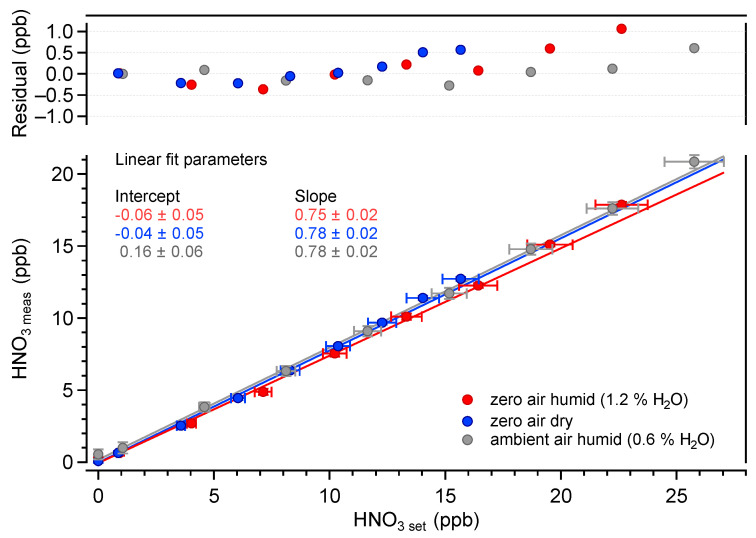
Measured HNO_3_ vs. calculated HNO_3_ amount fractions in various carrier gas conditions. The fit parameters are given with their absolute uncertainties (k = 2).

The measured HNO_3_ amount fractions as a function of the calculated set values are plotted in Figure 6. The set values were determined using the dilution factor (obtained from the mass flow values) and the HNO_3_ output amount fraction of the ReGaS. The latter value was measured using the liquid trapping/ion chromatography method, as described in Section 2.2. The mean value obtained over five measurements made during five consecutive days is 58.6±1.2 ppb (1σ). The error bars in Figure 6 show the repeatability standard deviation and do not take into account the uncertainties in the X and Y values (discussed below).

The linearity of the HNO_3_ measurements is excellent over the investigated range of amount fractions. In all conditions, $R^{2}$ is higher than 0.99. The average slope of the linear regressions is 0.78, while the intercept values are zero within the uncertainty of the measurements. The average slope value differs significantly from unity, indicating some systematic errors in either or both of the measured and set values. The uncertainty of the QCLAS data (Y axis) is dominated by the uncertainty of the absorption line strengths tabulated in the HITRAN database. A recent work reported line strength values that were 8% lower than those in HITRAN2020 for transitions of the ν2 band [40]. Using these values, the slope between the measured and set amount fractions becomes 0.9. The remaining 10% difference is well within the uncertainties in the calculated set values, which are largely dominated by the uncertainty of the determination of the ReGaS output amount fraction using liquid trapping (about 20%).

These linearity tests indicate the absence of chemical interference from H_2_O in HNO_3_ detection. To confirm this, complementary experiments were performed by sampling a constant amount of HNO_3_ in the presence of varying amounts of water vapor of up to 1.2% H_2_O. Each H_2_O amount fraction value was bracketed by a humid HNO_3_ blank measurement to account for possible H_2_O effects on the zero signal.

**Figure 7:**
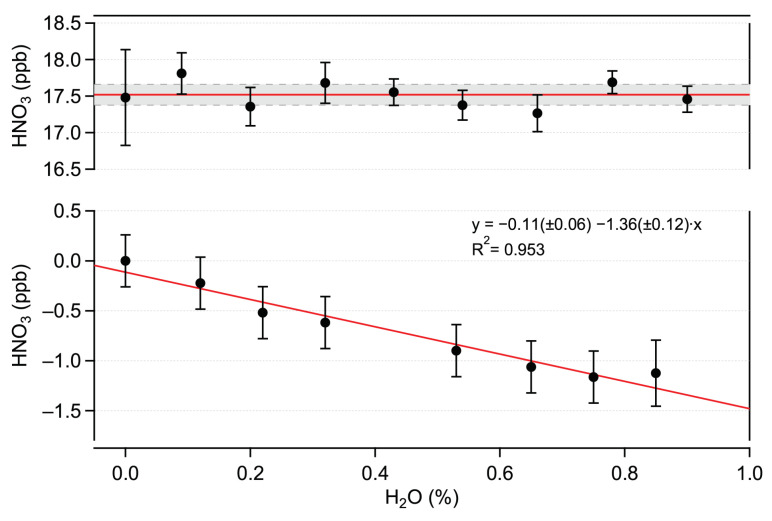
**Top**: offset-corrected HNO_3_ amount fractions measured in varying humidity content. The solid red line represents the mean value. The dashed lines indicate the standard deviation (±1σ). **Bottom**: influence of humidity on the HNO_3_ offset signal equivalent HNO_3_ amount fraction.

The offset-corrected HNO_3_ amount fractions measured at different humidity values are shown in the top panel of Figure 7. No significant dependency on the H_2_O amount fractions is observed. These data demonstrate the absence of the physico-chemical influence of water vapor on HNO_3_ measurements in the investigated ranges. However, H_2_O has a notable impact on the zero signal (offset), as shown in the bottom panel of Figure 7. A similar effect has been demonstrated for NO_2_ measurements in the mid-IR range made using QCLAS (e.g., [28,34]). The magnitude (about 1.40 ppb HNO_3_/% H_2_O) of this effect for HNO_3_ is much stronger (20×) than it is for NO_2_. This can be due to the higher line strengths of the H_2_O transitions in the 1720 cm${}^{-1}$ spectral region, by the lower spectral spacing between the HNO_3_ and H_2_O lines, and to the lower HNO_3_ line strength. This biasing effect on ambient air measurements is mitigated by the zero-drift-correction procedure (via HNO_3_ scrubbing), which makes it possible to obtain a “humid” zero signal (see Section 3.2 for more details).

### 3.2. Field Results

Assessing the accuracy of the QCLAS measurements in the field is difficult due to the lack of a reliable, time-resolved reference method. As an alternative, we perform two different comparisons and an analysis to evaluate our measurements. First, we compare the QCLAS measurements with the total nitrate data (ΣNO${}_{3}^{-}$) obtained using the filter method described in Section 2.3.2. Then, we correlate the spectroscopic measurements with the so-called NO_2_ “Mo-CLD bias” (hereafter ΔNO_2_). This term represents the difference between NO_2_ measured by the Mo-CLD and NO_2_ measured by the reference CAPS instrument, which is a more specific, interference-free technique.

**Figure 8:**
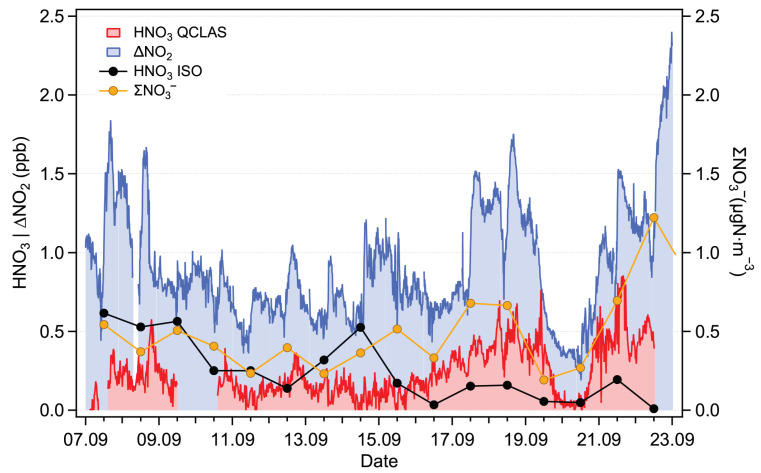
Time series of HNO_3_ measured by the QCLAS (red) and estimated using the ISORROPIA model (black), ΣNO${}_{3}^{-}$ (orange) and the difference in NO_2_ between the Mo-CLD and the CAPS instruments (ΔNO_2_, blue).

The time series for the campaign period (September 2021) are shown in Figure 8. The ΣNO${}_{3}^{-}$ data, given in μgN·m${}^{-3}$, are presented with the original resolution of 1 day (filters were changed every 24 h). The ΔNO_2_ values are plotted with a resolution of 10 min. The QCLAS data are given with a resolution of 15 min (corresponding to the mean value of the 8-min measurement period bracketed by two HNO_3_ blank measurements). As mentioned in Section 2.3.1, the HNO_3_ blank is obtained under ambient humidity conditions in order to mitigate spectroscopic interference caused by water (Section 3.1.2). However, a difference in the measured H_2_O amount fractions was observed between the ambient and zeroing channels in both laboratory and field conditions. This difference (about 0.02% H_2_O) was attributed to losses of H_2_O in the HNO_3_ scrubber of the zeroing channel. Using the data presented in the bottom panel in Figure 7, this difference can be converted into an HNO_3_ equivalent signal of approximately 30 ppt. A correction to account for this effect was applied to the data. We estimate a combined, mean relative uncertainty of 36% (k = 2) for the HNO_3_ field measurements. This combined uncertainty includes a relative uncertainty of 20% in the HNO_3_ calibration factor (accounting for the ReGaS instrument output and dilution setup), a relative uncertainty of 30% in the water bias correction factor (linear fit in Figure 7) and a conservatively estimated uncertainty of 10% in the measurement of the water concentration during the field campaign (obtained using spectroscopic and instrument parameters).

The ΣNO${}_{3}^{-}$ and the QCLAS data show similar trends, especially during the period between 15 and 22 September, when HNO_3_ amount fractions increase to about 0.8 ppb. The mismatch in the timing may be due to two independent factors: (i) the different response times of the two instruments’ inlets and (ii) the shares of gas phase HNO_3_ and particle phase NO${}_{3}^{-}$, which may vary in time and lead to a discrepancy between ΣNO${}_{3}^{-}$ and spectroscopically measured HNO_3_.

In an attempt to further evaluate our measurements, we used the ISORROPIA thermodynamic equilibrium model to estimate the gas phase HNO_3_ amount fraction [37]. The model is used in the SO${}_{4}^{2-}$, NH${}_{4}^{+}$, and NO${}_{3}^{-}$ modes. Minor species such as Na^+^, Cl${}^{-}$, and Mg^2+^ are not included since no direct measurements were available. Additionally, while NO${}_{3}^{-}$ and NH${}_{4}^{+}$ are well represented by the filter measurements, the SO${}_{4}^{2-}$ values only take into account the particle phase (and neglect the gas phase sulfuric acid (H_2_SO_4_)). Nevertheless, this constrained configuration is expected to be accurate enough to obtain a representative estimation of the HNO_3_ amount fraction. The results of the model calculation are plotted in Figure 8. Although the model predictions have the same order of magnitude as the QCLAS measurements, significant discrepancies are observed. For the second half of the measurement period, when the measured HNO_3_ is the highest, the model predictions are a factor of 2 lower than the measurements. Possible sources of error include the overestimation of the measured HNO_3_, underestimation of the total nitrates (up to 0.13 μgN·m${}^{-3}$), and underestimation of particle phase cation concentrations (which would displace the particle-phase-to-gas-phase NO${}_{3}^{-}$ equilibrium).

Previous studies showed that NO_2_ amount fractions measured by the Mo-CLD tend to be overestimated due to the dissociation of other nitrogen oxide compounds [41] in the converter. Similarly, a significant fraction of HNO_3_ was found to dissociate to NO_2_ [42], and thus, the Mo-CLD bias (ΔNO${}_{2}$) measured at the Rigi station is expected to correlate with the HNO_3_ amount fraction measured by our QCLAS. By investigating these correlations, two different situations were identified: the first one corresponds to the measurement period from 7 to 15 September, while the second is between 16–22 September. During the first period, no correlation is found (slope of 0.11 with an $R^{2}$ coefficient of 0.12), while for the second period, there is a correlation (slope of 0.38 with an $R^{2}$ coefficient of 0.6). This indicates that on some days, 40% of the Mo-CLD error (ΔNO${}_{2}$) can be explained by the dissociation of HNO_3_ in the molybdenum converter. The difference between the two periods is likely the result of changes in the HNO_3_-to-NO${}_{z}$ ratio. This ratio depends on the air mass “history”, and it is influenced by factors such as dry and wet deposition, VOC emissions, the ammonia amount fraction, and the photo-oxidation capacity of the atmosphere [42].

## 4. Conclusions

We have developed and characterized a mid-IR laser spectrometer for high-precision HNO_3_ measurements. The custom multipass cell with an optical path length of 110 m makes it possible to reach a precision of 140 ppt at a resolution of 1 s and a best precision of 20 ppt at a resolution of 2 min. The limit of detection of our spectrometer was estimated to be 95 ppt. We found that the passivation treatment of the aluminum surfaces, consisting of an electro-polishing step that is followed by coating the surface with a Silconert2000 layer, is comparable to the more often used fused silica and fluoro-silane-based combination. A response time of 2 min at a flow rate of 8.5 slpm for the multipass cell was achieved and no dependency on the absolute humidity (between 0 and 1%) was observed. We showed that the adsorption of HNO_3_ on PFA at low pressure is negligible, but that sampling at ambient pressure significantly increases the response time of the instrument. While no chemical interference from water has been detected (up to 1.2% absolute humidity), we found non-negligible spectroscopic interference that is mitigated by the measurement procedure. An excellent linearity was found between 0 and 25 ppb HNO_3_, with $R^{2}$ = 0.99, in both wet and dry conditions. The spectroscopically retrieved concentrations are 20% lower than the calculated reference gas concentrations (retrieved using liquid trapping and ion chromatography). This was attributed to the combined error of the HNO_3_ absorption line strength and the HNO_3_ concentration of the calibration method.

The QCLAS was deployed during a three-week field campaign in September 2021 in central Switzerland. The measured time series of the HNO_3_ amount fraction varied between 0.1 ppb and 0.8 ppb and correlated well with the daily total nitrates measured using a filter trapping method. Theoretically estimated HNO_3_ values were obtained using a thermodynamic equilibrium model and show a similar order of magnitude compared to our measurements. Finally, we considered the correlation of the retrieved HNO_3_ data with the difference between the measurement of NO_2_ by a CLD equipped with a molybdenum converter and by a selective, direct spectroscopic method (CAPS). We observed that on some days, 40% of the error (using the direct method as a reference) can be explained by the dissociation of HNO_3_ in the molybdenum converter.

## Data Availability

The data presented in this study are available on request from the corresponding author.

## Abbreviations

The following abbreviations are used in this manuscript:

ADC	analog-to-digital converter
CAPS	cavity-attenuated phase shift spectrometer/spectroscopy
CIMS	chemical ionization mass spectrometry
CLD	chemiluminescence detector/detection
CW	continuous wave
DAC	digital-to-analog converter
DAQ	data acquisition
DFB-QCL	distributed feedback quantum cascade laser
FPGA	field-programmable gate array
FSR	free spectral range
iCW	intermittent continuous wave
LAS	laser absorption spectroscopy
LOD	limit of detection
MCT	mercury cadmium telluride
MFC	mass flow controller
mid-IR	mid-infrared
Mo	molybdenum
MPC	multipass cell
OPL	optical path length
PCB	printed circuit board
PFA	perfluoroalkoxy alkane
ppb	part per billion by volume
ppt	part per trillion by volume
PTFE	polytetrafluoroethylene
QCLAS	quantum cascade laser absorption spectrometer/spectroscopy
TEC	thermoelectric cooling
VOCs	volatile organic compounds
